# The Novel Coronavirus Disease (COVID-19): A PRISMA Systematic Review and Meta-Analysis of Clinical and Paraclinical Characteristics

**DOI:** 10.1155/2020/3149020

**Published:** 2020-08-14

**Authors:** Hamidreza Hasani, Shayan Mardi, Sareh Shakerian, Nooshin Taherzadeh-Ghahfarokhi, Parham Mardi

**Affiliations:** ^1^Eye Research Center, The Five Senses Institute, Rasoul Akram Hospital, Iran University of Medical Sciences, Tehran, Iran; ^2^Department of Ophthalmology, Madani Hospital, Alborz University of Medical Sciences, Karaj, Iran; ^3^Student Research Committee, Alborz University of Medical Sciences, Karaj, Iran; ^4^Departments of Community Based Education of Health Sciences, Virtual School of Medical Education and Management, Shahid Beheshti University of Medical Sciences, Tehran, Iran

## Abstract

An outbreak of pneumonia, caused by a novel coronavirus (SARS-CoV-2), was identified in China in December 2019. This virus expanded worldwide, causing global concern. Although clinical, laboratory, and imaging features of COVID-19 are characterized in some observational studies, we undertook a systematic review and meta-analysis to assess the frequency of these features. We did a systematic review and meta-analysis using three databases to identify clinical, laboratory, and computerized tomography (CT) scanning features of rRT-PCR confirmed cases of COVID-19. Data for 3420 patients from 30 observational studies were included. Overall, the results showed that fever (84.2%, 95% CI 82.6-85.7), cough (62%, 95% CI 60-64), and fatigue (39.4%, 95% CI 37.2-41.6%) are the most prevalent symptoms in COVID-19 patients. Increased CRP level, decreased lymphocyte count, and increased D-dimer level were the most common laboratory findings. Among COVID-19 patients, 92% had a positive CT finding, most prevalently ground-glass opacification (GGO) (60%, 95% CI 58-62) and peripheral distribution opacification (64%, 95% CI 60-69). These results demonstrate the clinical, paraclinical, and imaging features of COVID-19.

## 1. Background

In December 2019, the first case of unknown origin pneumonia was identified in Wuhan, the capital city of Hubei Province. By January 7, 2020, Chinese scientists had isolated a novel virus belongs to coronaviruses family and classified it as a type of RNA virus [1].

Although the outbreak has been started from a primary zoonotic virus transmission in a large seafood market (Huanan Seafood Market), person-to-person transmission of the virus started a pandemic involving 197 countries [2, 3].

The clinical outcomes of SARS-CoV-2 infection are various, including asymptomatic infection, mild upper respiratory tract illness, severe viral pneumonia, and even death. Patients are presented with various clinical manifestations such as fever, dyspnea, and cough [4].

Initially, some studies have observed particular imaging patterns on chest radiography and computed tomography in COVID-19 patients [5]. As our knowledge increased, recent studies claimed that the sensitivity of CT scan is higher compared to rRT-PCR in the diagnosis of COVID-19 [6].

Laboratory findings are essential in order to evaluate patients' complications and triaging them [7]. Complete blood count as an easy and affordable test detects disorders such as leukopenia, anemia, and thrombocytopenia that are contributed to patients' prognosis [8]. In response to inflammation induced by COVID-19, acute-phase reactants may increase or decrease [9]. These factors may contribute to the patients' outcomes. This meta-analysis is aimed at measuring the most common clinical, laboratory, and imaging findings among COVID-19 patients.

## 2. Methods

### 2.1. Protocol

**Figure 1:**
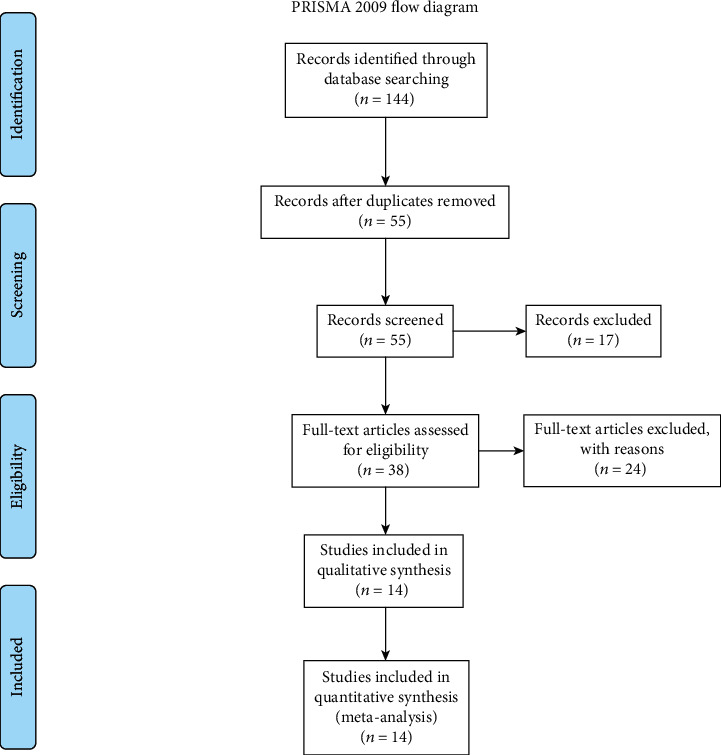
PRISMA flow chart of search, inclusion and exclusion screening, and accepted studies of the review on the clinical characteristics.

**Figure 2:**
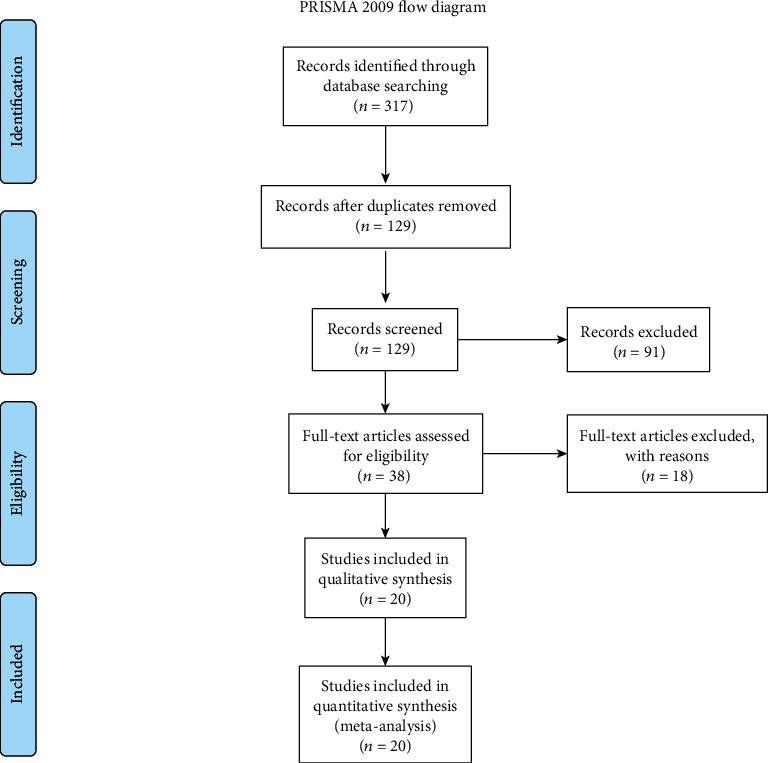
PRISMA flow chart of search, inclusion and exclusion screening, and accepted studies of the review on the imaging studies.

**Figure 3:**
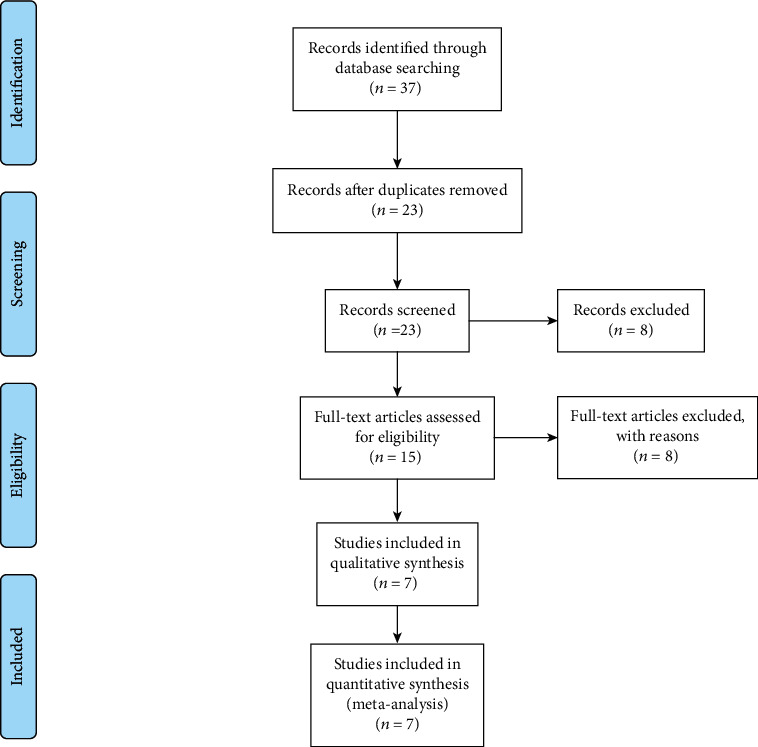
PRISMA flow chart of search, inclusion and exclusion screening, and accepted studies of the review on the laboratory findings.

In this study, we used a protocol based on the transparent reporting of systematic reviews and meta-analysis (PRISMA) (Figures 1–3).

### 2.2. Eligibility Criteria

In this study, all included patients were confirmed using real-time reverse transcriptase-polymerase chain reaction (rRT-PCR). All searched articles were cross-sectional studies, reporting descriptive data, and no language restrictions were conducted. All articles published before drafting the manuscript have been included. Review articles, opinion articles, and letters not presenting original data were excluded from the analysis.

### 2.3. Information Sources and Search Strategy

Three systematic searches were performed using Medline/PubMed, Scopus, and Web of Science. Systematic search was conducted prior to March 25, 2020, and three independent researchers evaluated all papers. The search was conducted based on the following keywords, and all studies were divided in three groups: (1) For clinical characteristics group: (“clinical manifestation” AND COVID-19) or (“clinical manifestation” AND 2019-nCoV) or (“clinical manifestation” AND COVID) or (“clinical manifestation” AND Corona) or (“clinical characteristics” AND COVID-19) or (“clinical characteristics” AND 2019-nCoV) or (“clinical characteristics” AND COVID) or (“clinical characteristics” AND corona). (2) For laboratory findings group: (liver AND COVID-19) or (liver AND 2019-nCoV) or (liver AND COVID) or (liver AND Corona) or (“blood gas” AND COVID-19) or (“blood gas” AND 2019-nCoV) or (“blood gas” AND COVID) or (“blood gas” AND corona). (3) For imaging studies group: (COVID-19 AND radiography) or (2019-nCoV AND radiography) or (Corona AND radiography) or (COVID AND radiography) or (COVID-19 AND radiographic) or (2019-nCoV AND radiographic) or (Corona AND radiographic) or (COVID AND radiographic) or (COVID-19 AND CT) or (2019-nCoV AND CT) or (Corona AND CT) or (COVID AND CT) or (COVID-19 AND “computed tomography”) or (2019-nCoV AND “computed tomography”) or (Corona AND “computed tomography”) or (COVID AND “computed tomography”) or (CBC AND corona) or (CBC AND COVID) or (CBC AND COVID-19) or (CBC AND 2019-nCoV).

### 2.4. Study Selection

In the initial search, we assessed the title and abstract, followed by a full-text evaluation based on previously described inclusion and exclusion criteria. When two articles reported one patient's characteristics, we merged all reported data and assumed as a single individual. Descriptive studies reporting clinical symptoms, laboratory, and radiological findings were used to perform a meta-analysis. The characteristics of the included studies are shown in Table 1. The modified appraisal tool for cross-sectional studies (AXIS) was used to determine the methodological quality of the research designs of the included studies (Table 2). AXIS is used to assess research papers systematically and to judge the reliability of the study being presented in the paper. It also helps in assessing the worth and relevance of the study. Studies with total scores of ten or less were excluded.

### 2.5. Data Collection Process and Data Items

Three independent researchers filled data extraction forms containing study type, journal, publication date, sample size, age, gender, clinical characteristics, laboratory, and radiological findings. Conflicts were resolved by another researcher.

### 2.6. Assessment of Methodological Quality and Risk of Bias

**Figure 4:**
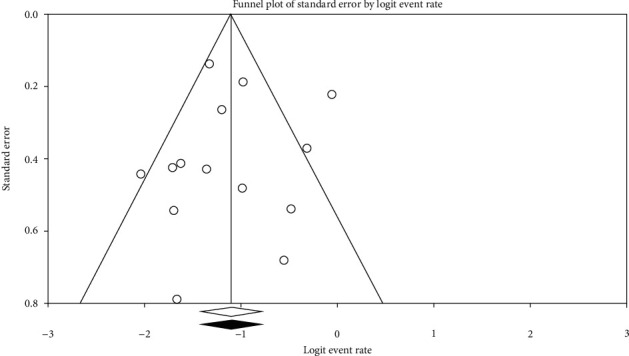
Funnel plot for clinical characteristics group studies.

**Figure 5:**
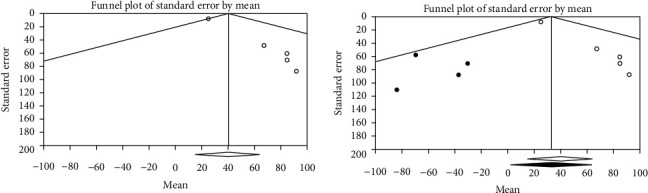
Funnel plots for observed studies and imputed studies (trim and fill test) of laboratory findings. The observed studies are shown as open circles, and the four imputed studies are shown as filled circles. The observed point estimate is shown as an open diamond, and the imputed point estimate is shown as a filled diamond.

**Figure 6:**
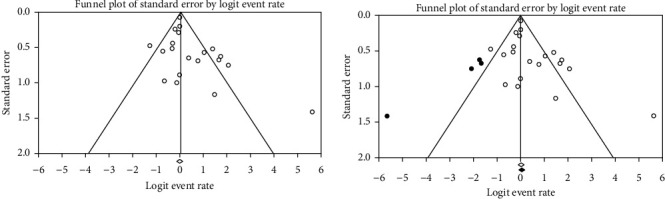
Funnel plots for observed studies and imputed studies (trim and fill test) of imaging studies. The observed studies are shown as open circles, and the four imputed studies are shown as filled circles. The observed point estimate is shown as an open diamond, and the imputed point estimate is shown as a filled diamond.

Publication bias was assessed with a funnel plot for the standard error and considering that the interpretation of the plot is subjective (Figures 4–6). Also, bias was quantified by using the Egger regression test.

**Figure 7:**
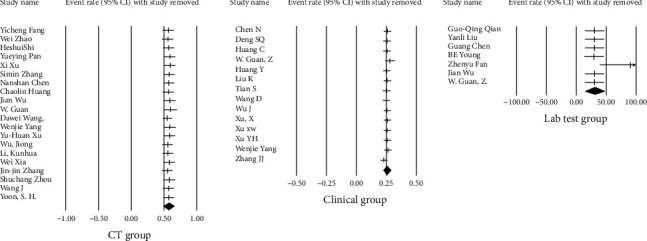
Sensitivity analysis of the meta-analysis for each of the included studies.

Sensitivity analysis and adjusting for risk bias were performed by the attractive test (trim and fill method). We initially identified and trimmed the asymmetric (missing) studies, followed by estimating the unbiased summary effect. Sensitivity analysis was also performed using the “Remove-One” analysis by running the analysis with each of the studies removed. The result of the impact of each study on the pooled estimate is shown in the forest plot (Figure 7).

The percentage of total variation across studies (heterogeneity) was measured by the inconsistency index tool (*I* squared). The *I* squared index measured for each of the clinical characteristics, imaging studies, and laboratory findings groups. *I* squared index value in the ranges of <25%, 25–50%, 50–75%, and >75% was interpreted as low, moderate, high, and very high heterogeneity, respectively [10].

**Figure 8:**
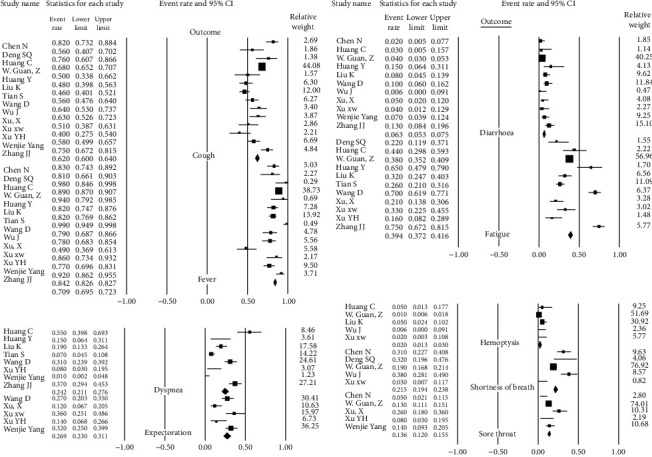
Pool prevalence forest plots of clinical manifestations.

**Figure 9:**
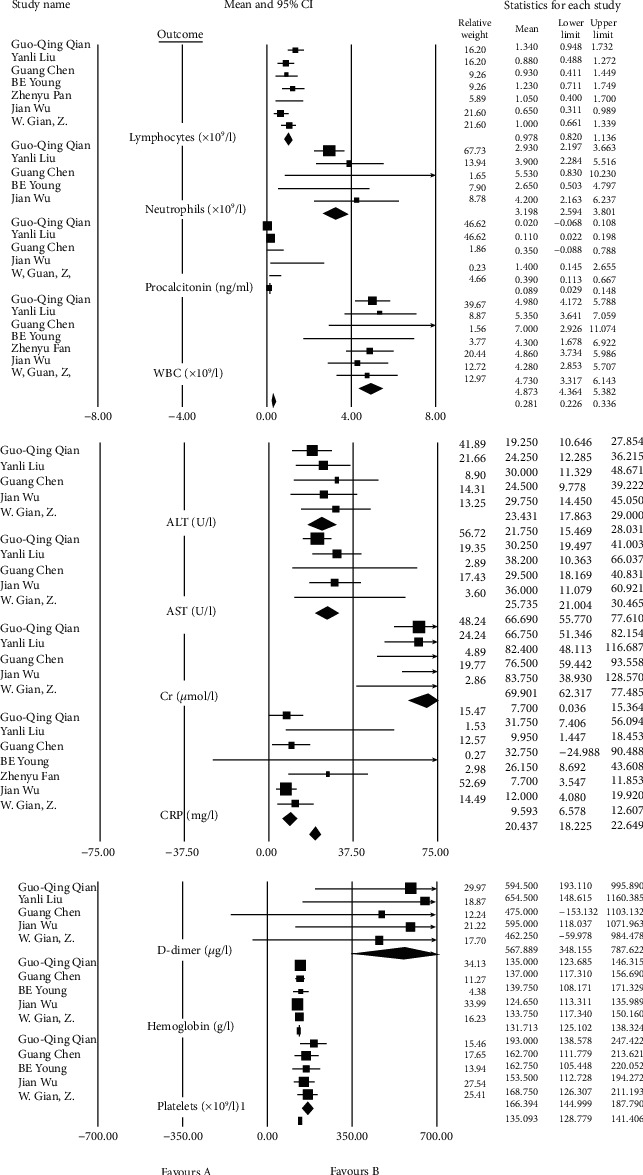
Pool prevalence forest plots of laboratory findings.

**Figure 10:**
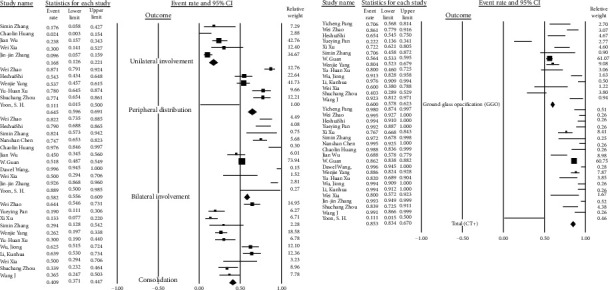
Pool prevalence forest plots of CT scan findings.

We conducted a random-effects analysis because it was assumed that some of the included studies did not share a common effect size (heterogeneity). Findings in each group are summarized as forest plots in Figures 8–10.

### 2.7. Statistical Approach

Effect size pooled estimate for imaging and clinical data was measured based on event rate, logit event rate, and standard error.

Considering that the computational index of laboratory data was median in order to meta-analyze them in CMA v.2. software, we use the following formula:

Estimating the mean and variance from the median:

(1) $$\begin{array}{c} S^{2}\approx \frac{1}{12}\left(\frac{{\left(a-2m+b\right)}^{2}}{4}+{\left(b-a\right)}^{2}\right),\\ \bar{x}\approx \frac{a+2m+b}{4}, \end{array}$$

where *m* is the median, *a* is the smallest value (minimum), *b* is the largest value (maximum), and *n* is the size of the sample [11].

The meta-analysis was performed using STATA, the software OpenMeta[Analyst], and Comprehensive Meta-Analysis Software (CMA) ve.2. Pooled estimate and their 95% confidence intervals (95% CIs) were used to summarize the weighted effect size for each study grouping variable.

## 3. Results

### 3.1. Study Selection and Characteristics

Two hundred seven articles were included based on a search strategy, which are previously described (Table 1). The full text of 65 articles was evaluated after the title and abstract assessment. Twenty-four articles were excluded due to inadequate data. Finally, the meta-analysis was performed on 30 articles (three different subjects). The article's data summary is reported in Table 2. Also, demographic characteristics and comorbidities of patients participated in the included studies are demonstrated in Table 3.

In this study, we evaluate 30 articles. All papers were from China, and 3420 individual's data were evaluated. All studies were cross-sectional, and 27 variables were included.

### 3.2. Heterogeneity

Evaluating the heterogeneity of the studies indicated that in the clinical characteristics and imaging studies groups, the combined effect of the *I* squared index is high (68.43, 68.53). While the laboratory findings groups combined effect of the *I* squared index is considered low (6.12). *I* squared index for each of the outcomes is shown in Tables 4–7.

### 3.3. Publication Bias and Sensitivity Analysis

The Funnel plot for clinical characteristics group studies is almost symmetric confirmed by the Egger regression test (intercept = −0.28, *P* value = 0.20). By using the random-effects model, the summary estimate and 95% confidence interval for the combined studies is 0.25 (0.22, 0.29). These findings indicated no publication bias in the clinical characteristics group (Figure 4).

The funnel plots in the imaging studies group and findings studies seem asymmetric and skewed (Figures 5 and 6). Also, the Egger regression test has indicated an intercept of 1.55, and *P* value of 0.01 for the laboratory findings group and an intercept of 0.94 and *P* value of 0.04 for the imaging studies group.

In the laboratory findings group, using the random-effects model, the point estimate and 95% confidence interval for the combined studies is 3.01 (2.22, 3.80). Using trim and fill (four trimmed studies), the imputed summary estimate is 3.30 (2.30, 3.38) (Figure 5).

In the imaging studies group, the summary estimate and 95% confidence interval for the combined studies is 0.51 (0.48, 0.54). Using trim and fill, the imputed (four trimmed studies) summary estimate is 0.50 (0.47, 0.53) (Figure 6).

In conclusion, the finding of trim and fill analysis has indicated only minimal changes, which do not seem to be a threat to the validity of the effect size estimates.

Sensitivity analysis by using the “Remove-One” analysis did not show any change in the combined effect of the clinical characteristics and imaging studies groups after removing any of the studies. However, in the laboratory findings group, removing only one of the studies (Fan et al.) changed the combined effect significantly (Figure 7). The combined effect before and after removing Fan et al. study is presented in Tables 5 and 6.

### 3.4. Clinical Characteristics Group

According to clinical manifestations, fever (84.3%, 95% CI 78.6-88.7), cough (60.1%, 95% CI 53.5-66.4), and fatigue (39.4%, 95% CI 29.1-50.8) are the most prevalent clinical symptoms among patients (Table 4) (Figure 8).

### 3.5. Laboratory Findings Group

Laboratory studies show increased level in following tests: CRP (10.78 mg/l, 95% CI 6.44-15.11) with the normal range of 0-3.0 mg/l, D-dimer (567.89 ng/ml, 95% CI 348.15-787.62) with the normal range of 0-500 ng/ml, LDH (258.56 U/l, 95% CI 206.84-310.29) with the normal range of 135-250 U/l, and procalcitonin (0.17 ng/ml, 95% CI 0.01-0.32) which is normally less than 0.05 ng/ml in a healthy individual.

Also, the level of some laboratory factors is lower than normal, such as lymphocyte (1.00∗10^9^/l, 95% CI 0.73-1.26) and albumin (38.61 g/l, 95% CI 35.75-41.48) concerning the normal range of 1‐4∗10^9^/l and 40-55 g/l, respectively (Tables 5 and 6) (Figure 9).

### 3.6. Imaging Studies Group

Among all patients infected by SARS-CoV-2 (confirmed by RT-PCR), 85% had abnormalities in CT scans. In most of them, the bilateral pneumonia was dominant (79.4%, 95% CI 66.9-88.1). Ground-glass opacification (GGO) (69.8%, 95% CI 60.3-77.9), peripheral distribution (66.8%, 95% CI 50.0-80.2), and consolidation (37.8%, 95% CI 26.4-50.8) in those with CT scan results are presented (Table 7) (Figure 10).

## 4. Discussion

From December 2019, more than 500,000 cases of new unknown origin pneumonia have been confirmed all over the world [12]. It was primarily known as 2019-nCov; then, WHO decided to name this novel coronavirus “SARS-CoV-2” [13]. COVID-19 is a severe condition that is compromising the health condition of people in all countries worldwide [14]. Identifying the various characteristics of this infection is vital for controlling the outbreak in different countries [15]. Clinical, laboratory, and imaging findings are essential to evaluate the different aspects of infection [16]. Different outcomes of COVID-19 (from an asymptomatic infection to death) and contagiousness of this virus, even in its incubation period [17], emphasize why discovering different characteristics are crucial in controlling this pandemic.

In this systematic review and meta-analysis, we describe the most common clinical data on COVID-19 confirmed cases that were published during the first months of the outbreak. We analyzed 2422, rRT-PCR confirmed patients, for different clinical manifestations. Our findings are robust due to the pooled results after combining all the studies' data.

As expected from initial studies in China, COVID-19 patients presented predominantly with cough and fever, as well as headache, diarrhea, and fatigue, among other clinical features [18]. This was consistently found in many of the included studies [19, 20]. Fever frequency is similar in other *β*-CoV-associated infections such as SARS and MERS, but studies showed that the cough frequency is higher in SARS and COVID-19 than MERS (<50%) [21, 22]. In SARS and MERS, diarrhea is reported in about a quarter of patients, but our data shows that only 6 percent of COVID-19 patients present with diarrhea (Table 4). Our data also suggest that about 11 percent of patients are presented with headache as a symptom. Unlike SARS, which is well characterized in the two-stage clinical course of the disease [23], COVID-19 still needs further definition to identify the disease process.

Studies on epidemiological features of COVID-19 showed that about 80 percent of patients are asymptomatic or are presented with mild manifestations [24, 25], but almost all of the patients included in our study had moderate-to-severe characteristics. It seems that fever and cough are the most common clinical features among moderate-to-severe patients (Table 4).

Studies show different laboratory abnormalities in COVID-19 patients, such as hypoalbuminemia or elevated inflammatory markers [26]. However, our data suggest that C-reactive protein is the most elevated factor among infected cases (Tables 5 and 6). D-dimer, LDH, and procalcitonin are also elevated in patients, which confirmed that measuring inflammatory markers are essential to investigate new cases [27]. Also, seven studies showed lymphopenia and albuminuria as other common laboratory findings. Data from new studies suggest lymphocytopenia or an increase in WBC as prognostic factors in COVID-19 patients. Studies on the SARS outbreak in 2003 indicate that lymphopenia, leukopenia, and thrombocytopenia, elevated levels of LDH, alanine transaminase (ALT), AST, and creatine-kinase are the most affected laboratory findings [28].

Nevertheless, not significantly seen in COVID-19, the novel corona virus can affect the liver and other organs [29]. AST and ALT are normal in most cases, but impaired liver function tests are associated with poor prognosis and higher mortality rates [30, 31]. Coagulation function tests (such as INR) are affected in the prognosis of this infection [32]. Lymphopenia in COVID-19 patients suggests that this virus might act on lymphocytes (mainly T cells), but some studies suggest that B cells are also affected [26, 33].

CT scan is one of the most useful methods to diagnose the respiratory tract diseases diagnosis. CT scan high sensitivity and availability makes it one of the most common tests for lung disease screening [34, 35]. In COVID-19 patients, different results could present in the early stages of infection [36, 37]; even some studies demonstrate that CT scan sensitivity is higher than rRT-PCR [6]. Our data shows that 92% of rRT-PCR confirmed cases had abnormal CT scan results, which suggest CT scan as a reliable method. As seen in Table 7, CT scan meta-analysis outcomes are performed in random-effects analyses. Our meta-analysis on 15 studies showed that ground-glass opacification (GGO) and peripheral distribution are seen on 69.8% and 66.8% of patients, respectively. 79.4% of the patients had bilateral involvements, which is contributed to poor prognosis. CT scan is useful in monitoring the treatment, and it is crucial in classifying patients and identifying who should be treated with aggressive treatments [38]. Other findings such as consolidation or reverse halo or atoll sign are reported in some studies [39], which were not included in our analysis.

## 5. Limitations

This review has several limitations. Few studies are available on COVID-19, and most of them are from China. Many countries such as Italy, the United States, and Iran reported several new COVID-19 patients, but data about clinical characteristics or laboratory findings are limited. By publishing more studies worldwide, researchers are going to get a more comprehensive understanding of COVID-19. Patients' detailed information, especially in clinical outcomes, was unavailable in most studies at the time of analysis. In this study, we used random-effects model for analysis in all three groups. In comparison to the fixed model, random model findings have wider confidence intervals and less accurate results, although heterogeneity in included studies could not be considered in the fixed model.

## 6. Conclusion

COVID-19 presents in the majority of cases with fever and cough. Laboratory findings such as elevated inflammatory markers can assist the diagnosis. Other laboratory indices, such as AST, ALT, or INR, are also affected in these patients. 92% of the RT-PCR confirmed that patients have abnormalities in CT scan most frequently bilateral involvement. Additional research with higher sample sizes is needed in order to describe the patients' characteristics more precisely.

## Figures and Tables

**Table 1:** Characteristics of the included studies.

Row	Author	Journal	Type	Date	Country	Sample size	Reference
Imaging							
1	Fang et al.	*Radiology*	Cross-sectional	Feb 19	China	51	[6]
2	Zhao et al.	*American Journal of Roentgenology*	Cross-sectional	Feb 18	China	101	[38]
3	Shi et al.	*The Lancet*	Cross-sectional	Feb 24	China	81	[40]
4	Pan et al.	*European Radiology*	Cross-sectional	Feb 13	China	63	[41]
5	Xu et al.	*European Journal of Nuclear Medicine and Molecular Imaging*	Cross-sectional	Feb 28	China	90	[42]
6	Zhang et al.	*European Respiratory Journal*	Cross-sectional	Mar 25	China	17	[43]
7	Chen et al.	*The Lancet*	Cross-sectional	Jan 30	China	99	[44]
8	Huang	*The Lancet*	Cross-sectional	Feb 21	China	41	[18]
9	Wu et al.	*Clinical Infectious Diseases*	Cross-sectional	Feb 29	China	80	[19]
10	Guan et al.	*The New England Journal of Medicine*	Cross-sectional	Feb 28	China	1099	[45]
11	Wang et al.	*Clinical Infectious Diseases*	Cross-sectional	Feb 29	China	138	[46]
12	Yang et al.	*Journal of Infection*	Cross-sectional	Feb 26	China	149	[47]
13	Xu et al.	*Journal of Infection*	Cross-sectional	Feb 25	China	50	[48]
14	Wu et al.	*Investigative Radiology*	Cross-sectional	Feb 29	China	80	[49]
15	Li et al.	*Investigative Radiology*	Cross-sectional	Feb 29	China	83	[50]
16	Xia et al.	*Pediatric Pulmonology*	Cross-sectional	Mar 05	China	20	[51]
17	Zhang et al.	*Allergy*	Cross-sectional	Feb 19	China	140	[52]
18	Zhou et al.	*American Journal of Roentgenology*	Cross-sectional	Feb 16	China	62	[53]
19	Wang et al.	*Journal of Zhejiang University*	Cross-sectional	Feb 24	China	52	[54]
20	Yoon et al.	*Korean J Radiol*	Cross-sectional	Apr 21	China	9	[55]
Laboratory							
21	Qian et al.	*An International Journal of Medicine*	Cross-sectional	Feb 21	China	91	[56]
22	Liu et al.	*medRxiv*	Cross-sectional	Feb 21	China	109	[57]
23	Chen et al.	*medRxiv*	Cross-sectional	Feb 14	China	21	[58]
24	Young et al.	*Jama*	Cross-sectional	Feb 24	China	18	[59]
25	Fan et al.	*The Lancet*	Cross-sectional	Mar 05	China	148	[30]
26	Wu et al.	*Clinical Infectious Diseases*	Cross-sectional	Feb 29	China	80	[19]
27	Guan et al.	*New England Journal of Medicine*	Cross-sectional	Feb 28	China	1099	[45]
Clinical characteristics							
28	Chen et al.	*The Lancet*	Cross-sectional	Feb 21	China	99	[44]
29	Deng and Peng	*Journal Clinical Medicine*	Cross-sectional	Feb 14	China	41	[60]
30	Huang	*Clinical Gastroenterology and Hepatology*	Cross-sectional	Feb 21	China	41	[18]
31	Guan et al.	*The New England Journal of Medicine*	Cross-sectional	Feb 19	China	1099	[45]
32	Huang et al.	*Travel Medicine and Infectious Disease*	Cross-sectional	Feb 24	China	34	[61]
33	Kui et al.	*Chinese Medical Journal*	Cross-sectional	Feb 07	China	137	[62]
34	Tian et al.	*Journal of Infection*	Cross-sectional	Feb 27	China	262	[63]
35	Wang et al.	*Jama*	Cross-sectional	Feb 21	China	138	[46]
36	Wu et al.	*Clinical Infectious Diseases*	Cross-sectional	Feb 29	China	80	[19]
37	Xu et al.	*European Journal of Nuclear Medicine and Molecular Imaging*	Cross-sectional	Feb 28	China	90	[42]
38	Xiao-Wei et al.	*BMJ: British Medical Journal*	Cross-sectional	Feb 19	China	62	[64]
39	Xu et al.	*Journal of Infection*	Cross-sectional	Feb 25	China	50	[48]
40	Yang et al.	*Journal of Infection*	Cross-sectional	Feb 26	China	149	[47]
41	Zhang et al.	*Allergy*	Cross-sectional	Feb 19	China	140	[52]

**Table 2:** Appraisal tool for cross-sectional studies (AXIS) scores of included studies.

Row	Author	Date	AXIS score						Reference
			Introduction	Methods	Results	Discussion	Others	Total	
1	Fang et al.	Feb 19	1	6	4	2	2	15	[6]
2	Zhao et al.	Feb 18	1	8	3	2	2	16	[38]
3	Shi et al.	Feb 24	1	9	3	2	2	17	[40]
4	Pan et al.	Feb 13	1	7	2	2	2	14	[41]
5	Xu et al.	Feb 28	1	6	4	2	2	15	[42]
6	Zhang et al.	Mar 25	1	7	2	2	2	14	[43]
7	Chen et al.	Jan 30	1	8	5	2	2	18	[44]
8	Huang	Feb 21	1	8	5	2	2	18	[18]
9	Wu et al.	Feb 29	1	6	2	2	1	12	[19]
10	Guan et al.	Feb 28	1	9	5	2	2	19	[45]
11	Wang et al.	Feb 29	1	9	5	2	2	19	[46]
12	Yang et al.	Feb 26	1	5	3	2	2	13	[47]
13	Xu et al.	Feb 25	1	6	4	2	2	15	[48]
14	Wu et al.	Feb 29	1	9	3	2	2	17	[49]
15	Li et al.	Feb 29	1	8	5	2	2	18	[50]
16	Xia et al.	Mar 05	1	5	2	2	2	12	[51]
17	Zhang et al.	Feb 19	1	6	2	1	2	12	[52]
18	Zhou et al.	Feb 16	1	5	4	2	2	14	[53]
19	Wang et al.	Feb 24	1	7	3	2	2	15	[54]
20	Yoon et al.	Apr 21	1	4	3	2	2	12	[55]
21	Qian et al.	Feb 21	1	7	4	2	2	16	[56]
22	Liu et al.		1	6	4	2	2	15	[57]
23	Chen et al.		1	5	2	2	2	12	[58]
24	Young et al.	Feb 24	1	8	4	2	2	17	[59]
25	Fan et al.	Mar 05	1	8	3	2	2	16	[30]
29	Deng and Peng	Feb 14	1	5	5	2	2	15	[60]
32	Huang et al.	Feb 24	1	5	3	2	2	13	[61]
33	Kui et al.	Feb 07	1	5	3	2	2	13	[62]
34	Tian et al.	Feb 27	1	5	3	2	2	13	[63]
38	Xiao-Wei et al.	Feb 19	1	7	2	2	2	14	[64]

**Table 3:** Demographical characteristics and comorbidities of patients in the included studies.

Row	Author	Date	Sample size	Mean age (y. old)	Age range	Sex (male)	Diabetes	Hypertension	Cardiovascular disease	COPD	Malignancies	Digestive system disease
1	Fang et al.	Feb 19	51	45	39-55	29	—	—	—	—	—	—
2	Zhao et al.	Feb 18	101	44/44	17-75	56	—	—	15/8	4/9	—	—
3	Shi et al.	Feb 24	81	49/5	39-61	42	12	15	10	11	5	9
4	Pan et al.	Feb 13	63	44/9	31-62	33	—	—	—	—	—	—
5	Xu et al.	Feb 28	90	50	18-86	39	6	19	3	1	2	2
6	Zhang et al.	Mar 25	17	48/6	23-74	8	—	11/7	5	11	—	11/7
7	Chen et al.	Jan 30	99	55/5	21-82	67	—	—	40	1	1	11
8	Huang	Feb 21	41	49	41-58	30	20	15	15	2	2	2
9	Wu et al.	Feb 29	80	46	18-65	39	—	—	31/25	1/25	1/25	3/75
10	Guan et al.	Feb 28	1096	49	35-58	637	7/4	15	3/9	1/1	0/9	2/1
11	Wang et al.	Feb 29	138	56	42-68	75	10/1	31/2	14/5	2/9	7/2	2/9
12	Yang et al.	Feb 26	149	45/1	30-68	81	—	—	18/79	0/67	1/34	5/37
13	Xu et al.	Feb 25	50	43	3-85	29	—	—	—	—	—	—
14	Wu et al.	Feb 29	80	44	30-52	42	5	5	1	4	—	—
15	Li et al.	Feb 29	83	45/5	25-64	44	7/8	6	1/2	6	—	—
16	Xia et al.	Mar 05	20	1	0-7	13	—	—	—	—	—	—
17	Zhang et al.	Feb 19	135	57	25-87	71	12	30	12/1	2/8	—	10/7
18	Zhou et al.	Feb 16	62	52/8	30-77	39	6	6	1	—	—	—
19	Wang et al.	Feb 24	52	—	—	—	—	—	—	—	—	—
20	Yoon et al.	Apr 21	9	54	—	—	—	—	—	—	—	—
21	Qian et al.	Feb 21	91	50	36-57	37	8/79	16/48	3/3	—	—	—
22	Liu et al.		109	55	43-66	59	11	33	6/4	3/7	—	—
23	Chen et al.		21	56	—	17	14/3	23/8	—	—	—	—
24	Young et al.	Feb 24	18	47	31-73	9	—	—	—	—	—	—
25	Fan et al.	Mar 05	148	50	36-64	73	—	—	—	—	—	—
26	Xu et al.	Feb 19	62	41	32-52	36	2	8	2	2	—	11
27	Deng and Peng	Feb 14	41	55/5	25-89		42/3	53/8	19/2	19/2	—	—
28	Huang et al.	Feb 24	34	56/24	26-88	14	11/8	23/5	17/6	2/9	8/8	2/9
29	Kui et al.	Feb 07	137	57	20-83	61	9/5	10/2	7/3	1/5	1/5	—
30	Tian et al.	Feb 27	262	47/5	1-94	127	—	—	—	—	—	—

**Table 4:** A meta-analysis of clinical characteristics group.

Clinical group		Effect size and 95% confidence interval			Test of null (2-tail)		Heterogeneity
Outcome	Number of studies	Point estimate	Lower limit	Upper limit	*Z* value	*P* value	*I* squared
Cough	14	0.601	0.535	0.664	2.975	0.003	87.304
Fever	14	0.843	0.786	0.887	8.650	0.000	87.006
Headache	12	0.091	0.070	0.118	-15.933	0.000	57.760
Diarrhea	11	0.064	0.043	0.095	-12.258	0.000	71.281
Fatigue	11	0.394	0.291	0.508	-1.827	0.068	94.073
Dyspnea	8	0.171	0.091	0.298	-4.290	0.000	92.769
Expectoration	5	0.239	0.164	0.334	-4.840	0.000	77.659
Hemoptysis	5	0.023	0.009	0.057	-7.652	0.000	70.741
Shortness of breath	5	0.241	0.151	0.361	-3.898	0.000	87.758
Sore throat	5	0.130	0.085	0.193	-7.866	0.000	78.329
Muscle ache	4	0.114	0.052	0.231	-4.749	0.000	83.406
Nausea	2	0.109	0.038	0.275	-3.633	0.000	81.783

**Table 5:** Meta-analysis of laboratory findings before removing Fan et al. study.

Group LAB test		Effect size and 95% confidence interval					Test of null (2-tail)		Heterogeneity
Outcome	Number of studies	Point estimate	Standard error	Variance	Lower limit	Upper limit	*Z* value	*P* value	*I* squared
CRP (mg/l)	7	10.78	2.21	4.89	6.44	15.11	4.87	0.0000	30.63
Lymphocytes (×10^9^/l)	7	1.00	0.13	0.02	0.73	1.26	7.48	0.0000	45.87
WBC (×10^9^/l)	7	4.87	0.26	0.07	4.36	5.38	18.76	0.0000	0.00
ALT (U/l)	5	23.43	2.84	8.07	17.86	29.00	8.25	0.0000	0.00
AST (U/l)	5	25.84	2.47	6.08	21.01	30.68	10.48	0.0000	1.71
Cr (*μ*mol/l)	5	69.90	3.87	14.97	62.32	77.48	18.06	0.0000	0.00
D-dimer (*μ*g/l)	5	567.89	112.11	12568.89	348.15	787.62	5.07	0.0000	0.00
Hemoglobin (g/l)	5	131.71	3.37	11.38	125.10	138.32	39.05	0.0000	0.00
LDH (U/l)	5	258.56	26.39	696.51	206.84	310.29	9.80	0.0000	11.06
Neutrophils (×10^9^/l)	5	3.20	0.31	0.09	2.59	3.80	10.38	0.0000	0.00
Platelets (×10^9^/l)	5	166.39	10.92	119.17	145.00	187.79	15.24	0.0000	0.00
Procalcitonin (ng/ml)	5	0.17	0.08	0.01	0.01	0.32	2.15	0.0312	68.47
Creatine kinase (U/l)	4	110.18	19.92	396.72	71.14	149.22	5.53	0.0000	0.00
Total bilirubin (mmol/l)	4	8.36	1.08	1.16	6.25	10.48	7.76	0.0000	0.00
Albumin (g/l)	3	38.61	1.46	2.14	35.75	41.48	26.41	0.0000	44.52
BUN (mmol/l)	3	4.83	0.55	0.30	3.75	5.91	8.77	0.0000	0.00
ESR	2	40.79	24.93	621.75	-8.08	89.67	1.64	0.1018	91.15
Fibrinogen (g/l)	2	3.21	0.22	0.05	2.78	3.64	14.71	0.0000	0.00

**Table 6:** Meta-analysis of laboratory findings after removing Fan et al. study.

Laboratory findings group (one study removed)		Effect size and 95% confidence interval			Test of null (2-tail)		Heterogeneity
Outcome	Number of studies	Point estimate	Lower limit	Upper limit	*Z* value	*P* value	*I* squared
CRP (mg/l)	6	9.13	6.00	12.27	5.71	0.00	1.73
Lymphocytes (×10^9^/l)	6	0.99	0.78	1.20	9.20	0.00	37.93
WBC (×10^9^/l)	6	4.88	4.31	5.45	16.75	0.00	0.00
ALT (U/l)	5	23.43	17.86	29.00	8.25	0.00	0.00
AST (U/l)	5	25.84	21.01	30.68	10.48	0.00	1.71
Cr (*μ*mol/l)	5	69.90	62.32	77.48	18.06	0.00	0.00
D-dimer (*μ*g/l)	5	567.89	348.15	787.62	5.07	0.00	0.00
Hemoglobin (g/l)	5	131.71	125.10	138.32	39.05	0.00	0.00
LDH (U/l)	5	258.56	206.84	310.29	9.80	0.00	11.06
Neutrophils (×10^9^/l)	5	3.20	2.59	3.80	10.38	0.00	0.00
Platelets (×10^9^/l)	5	166.39	145.00	187.79	15.24	0.00	0.00
Procalcitonin (ng/ml)	5	0.17	0.01	0.32	2.15	0.03	68.47
Creatine kinase (U/l)	4	110.18	71.14	149.22	5.53	0.00	0.00
Total bilirubin (mmol/l)	4	8.36	6.25	10.48	7.76	0.00	0.00
Albumin (g/l)	3	38.61	35.75	41.48	26.41	0.00	44.52
BUN (mmol/l)	3	4.83	3.75	5.91	8.77	0.00	0.00
Fibrinogen (g/l)	2	3.21	2.78	3.64	14.71	0.00	0.00

**Table 7:** A meta-analysis of imaging study outcomes.

CT group	Number of studies	Effect size and 95% confidence interval			Test of null (2-tail)		Heterogeneity
Outcome		Point estimate	Lower limit	Upper limit	*Z* value	*P* value	*I* squared
Total (CT+)	20	0.923	0.877	0.953	9.290	0.000	81.398
(1) Ground-glass opacification (GGO)	14	0.698	0.603	0.779	3.890	0.000	90.700
(4) Bilateral involvement	11	0.794	0.669	0.881	4.081	0.000	93.032
(5) Consolidation	11	0.378	0.264	0.508	-1.845	0.065	90.564
(3) Peripheral distribution	6	0.668	0.500	0.802	1.955	0.051	88.951
(10) Unilateral involvement	5	0.156	0.082	0.278	-4.510	0.000	71.765
(∗∗) Mixed opacity	4	0.328	0.121	0.635	-1.106	0.269	97.487
(11) Air bronchogram	4	0.426	0.225	0.656	-0.618	0.537	87.500
(6) Pleural effusion	4	0.066	0.036	0.120	-7.950	0.000	14.830
(7) Adjacent pleura thickening	4	0.409	0.211	0.641	-0.764	0.445	92.842
(12) Fibrous stripes	3	0.229	0.056	0.596	-1.486	0.137	93.828
(2) Lower lung predominant	3	0.624	0.467	0.758	1.558	0.119	64.196
(8) Interlobular septal thickening	3	0.535	0.359	0.703	0.379	0.705	85.123
1 lobe affected	2	0.207	0.087	0.417	-2.608	0.009	83.897
2 lobes affected	2	0.061	0.032	0.113	-7.927	0.000	0.000
3 lobes affected	2	0.105	0.047	0.220	-4.796	0.000	57.131
4 lobes affected	2	0.099	0.060	0.157	-8.135	0.000	0.000
5 lobes affected	2	0.394	0.312	0.483	-2.331	0.020	18.313
(9) Cavitation	2	0.012	0.002	0.082	-4.350	0.000	0.000
Crazy-paving pattern	2	0.222	0.067	0.530	-1.786	0.074	92.080
Linear opacities	2	0.630	0.555	0.699	3.372	0.001	0.000

## Abbreviations

COVID-19: Corona virus disease of 2019
rRT-PCR: Real-time reverse transcription-polymerase chain reaction
CT scan: Computed tomography scan
CI: Confidence intervals
CRP: C-reactive protein
RNA: Ribonucleic acid
SARS-CoV-2: Severe acute respiratory syndrome corona virus #2
PRISMA: Protocol based on the transparent reporting of systematic reviews and meta-analysis
CBC: Complete blood count
CMA: Comprehensive meta-analysis
GGO: Ground-glass opacification
*β*-CoV: Beta coronavirus
MERS: Middle East respiratory syndrome
SARS: Severe acute respiratory syndrome
LDH: Lactate dehydrogenase
WBC: White blood cell
ALT: Alanine transaminase
AST: Aspartate aminotransferase
INR: International normalized ratio.
